# The steady-state level of plasma membrane ceramide is regulated by neutral sphingomyelinase 2

**DOI:** 10.1016/j.jlr.2024.100719

**Published:** 2024-12-02

**Authors:** Anne G. Ostermeyer-Fay, Abhay Kanodia, Ranjana Pathak, Maria Jose Hernandez-Corbacho, Aarnoud C. van der Spoel, Yusuf A. Hannun, Daniel Canals

**Affiliations:** 1 Department of Medicine, Cancer Center at Stony Brook, Stony Brook, NY, USA; 2 Graduate Program in Genetics, Stony Brook University, Stony Brook, NY, USA; 3 The Atlantic Research Centre, Department of Biochemistry & Molecular Biology, Dalhousie University, Halifax, Nova Scotia, Canada; 4 Department of Biochemistry and Cell Biology, Stony Brook University, Stony Brook, NY, USA; 5 Department of Pathology, Stony Brook University, Stony Brook, NY, USA; 6 Biological Mass Spectrometry Center, Stony Brook University, Stony Brook, NY, USA

**Keywords:** neutral sphingomyelinase 2, acid sphingomyelinase, GBA2, sphingomyelin synthase 2, neutral ceramidase, acid ceramidase, cellular compartmentalization, plasma membrane ceramide, ceramide, sphingomyelin, sphingolipids, subcellular organelles, confluence

## Abstract

During the last 30 years, an increasing number of cellular functions have been reported to be regulated by the lipid ceramide. The diversity in the ceramide structure, leading to tens of ceramide species and the discrete distribution based on subcellular topology, could explain the wide variety of functions attributed to this bioactive lipid. One of these pools of ceramide resides in the plasma membrane, and several works have suggested that an increase in plasma membrane ceramide (PMCer) in response to stimulation leads to cell death and modulates cell adhesion and migration. However, there is a limitation in studying PMCer content in this location primarily due to the inability to quantify its mass. Our group recently developed a method to specifically quantitate PMCer. In this work, we interrogate what sphingolipid metabolizing enzymes are responsible for modulating the basal levels of plasma membrane ceramide. An *in-silico* prediction and experimental confirmation found an almost perfect correlation between the endogenous expression levels of neutral sphingomyelinase (nSMase2) and the amount of plasma membrane ceramide in unstimulated cells. Manipulating the expression levels of nSMase2, but not other candidate enzymes of ceramide metabolism, profoundly affected PMCer. Moreover, a physiologic induction of nSMase2 during cell confluence resulted in a nSMase2-dependent dramatic increase in PMCer. Together, these results identify nSMase2 as the primary enzyme to regulate plasma membrane ceramide.

Several pieces of evidence suggest that ceramide signaling is not a unique linear pathway but a complex network with distinct pools of ceramide. Studies on ceramide signaling showed that ceramide generated in or transported to different membranes mediates different biological functions (1). For example, ceramide in mitochondria resulted in mitophagy (2) and apoptotic cell death (3); generation of ceramide in the Golgi apparatus triggered Golgi fragmentation (4); transport of ceramide from ER to multivesicular endosomes initiates extracellular vesicle secretion (5); and ceramide in the plasma membrane (PMCer) was involved in the modulation of cell adhesion and cell migration (6). Other biological processes, such as cell differentiation (7) and cell cycle arrest (8), are amongst the most studied in ceramide signaling (9), but the pool of ceramide involved is not well-established (10).

In an attempt to understand sphingolipid topology, targeted constructs to localize ceramide metabolizing enzymes to different membranes were designed and reported. These included the nuclear membrane, ER, Golgi, mitochondria, and plasma membrane (11). This study revealed that the content and composition of ceramide species are different in different membranes. Following this work, we suggested that the ceramide content in the plasma membrane in HeLa cells was very low, but it increased in response to stimulation with doxorubicin (12), matching the expected behaviors for a bioactive lipid. Thus, increased PMCer was necessary for cell migration in response to the drug. When PMCer was generated artificially, it was also sufficient to trigger cell migration without additional stimulation. Mechanistically, PMCer triggered the dephosphorylation of a network of proteins that controls the cortical cytoskeleton required for cell migration (6). We and other authors have implicated PMCer in TNFα and Fas-mediated cell death (13), mitochondrial fusion (14), and plasma membrane repair (15, 16). In addition, other works claim that ceramide microdomains in the plasma membrane (often also called rafts or ceramide-rich platforms) trigger biological functions by clustering and activating membrane receptors. For example, these microdomains have been reported to be necessary and sufficient to trigger cell death (17, 18). These results indicate a critical need to study the plasma membrane pool of ceramide. The primary current tool to detect plasma membrane ceramide is the use of ceramide antibodies. However, ceramide antibodies provide only qualitative information and cannot be used for quantification or estimating the mass of PMCer (19). Many of these antibodies are also not highly specific for ceramide either (20). Accordingly, we recently developed a quantitative method for the measurement of PMCer. This method optimized for adherent cells is very simple and highly sensitive, and for the first time, we can quantify the generation of ceramide at the plasma membrane in basal conditions and upon stimulation (21) in the context of ceramide compartmentalization (22).

In the current work, we used this novel method to interrogate what enzymes are responsible for maintaining a steady state PMCer pool. Mammalian cells contain several sets of enzymes that metabolize ceramide (23). These enzymes are localized in different membranes. However, the enzymes that generate ceramide at the plasma membrane are poorly defined. For example, their action at the plasma membrane is based on their subcellular localization, but many of these enzymes localize in multiple locations, making it difficult to evaluate if changes in ceramide occur in one or several of these membranes (9). Moreover, lipid transfer proteins and vesicular transport also contribute to the re-distribution of ceramide between membranes. The contribution of each of these enzymes to plasma membrane generation has not been explored.

Our approaches from this work were able to quantify PMCer in distinct cell lines, showing they are highly variable, but controlled by neutral sphingomyelinase 2 (nSMase2). Among the different enzymes tested, nSMase2 was the only one that significantly regulated ceramide levels at the plasma membrane. Other sphingolipid-metabolizing enzymes reported to localize to the plasma membrane had no or minimal effect on PMCer levels. Moreover, cell confluence, a known physiological inducer of nSMase2 transcription, also increased PMCer. These results demonstrate that intracellular levels of nSMase2 regulate the steady-state levels of PMCer. In addition, we found an almost perfect correlation between PMCer levels and nSMase2 expression across different cell lines. In summary, we identified nSMase2 as the primary enzyme to regulate steady-state PMCer.

## Material and Methods

### Cell lines

HeLa (CRM-CCL-2, epithelial cell from cervical adenocarcinoma), MCF-7 (HTB-22, epithelial from breast adenocarcinoma), HCC827 (lung adenocarcinoma), HT29 (Colorectal adenocarcinoma), HCT116 (colorectal carcinoma), PC3 (prostate adenocarcinoma), SW620 (Colorectal adenocarcinoma), MiaPaCa (pancreas carcinoma) and BT20 cells (Breast carcinoma) were purchased at ATCC and maintained in the recommended media from ATCC. Media included Dulbecco's Modified Eagle Medium high glucose from Thermo Fisher, Eagle's Minimum Essential Medium (EMEM), and RPMI 1640 medium. All media were supplemented with 10% of fetal bovine serum (Thermo Fisher). All cell lines were tested for mycoplasma on a monthly base. Cells were cultivated in a humidified incubator at 37°C in a 5% CO_2_ atmosphere.

### PMCer quantification

PMCer was measured as recently reported (21). Briefly, cells were plated on a 60 mm-diameter cell culture dish at 5 × 10^5^ cells per dish. The next day, the cells were washed with serum-free media and grown overnight in serum-free media at 37°C in a 5% CO_2_ atmosphere. On the third day, media were removed, and the cells were fixed with 4% paraformaldehyde (in PBS) for 20 min at room temperature. Parallel dishes were then washed with serum-free media and treated with or without purified pCDase. The dishes were incubated for 1 h at 37°C in a 5% CO_2_ atmosphere. Following, the dishes were washed with serum-free media, and LC-MS measured pmols/samples of sphingosine. PMCer was calculated as the arithmetic subtraction between the pmols of sphingosine with and without pCDase treatment. The amount of PMCer can be then normalized by the cell amount by quantifying inorganic phosphate as described in Materials and Methods.

### Protein purification

Bacteria *E. coli* BL21(DE3) pLysS competent cell strain (Stratagene) transfected with pET28 expressing his-tagged fusion *Bacillus cereus* sphingomyelinase C and *Pseudomonas aeruginosa* ceramidase plasmids were from previous studies from our group (24). Bacteria were grown in Luria-Bertani broth (LB) containing selective antibiotics. Cells were monitored at OD600, and 1 L of culture was induced with 1 mM of Isopropyl β-D-1-thiogalactopyranoside (IPTG) for protein expression when cells reached OD600 of 0.7. Cells were cultured at 30°C for 24 h and pelleted at 5000 *g*. Each protein was purified using affinity chromatography with a HisTrap FF 5 ml column and size-exclusion column (Superdex 200 HiScale 26/40) using an FPLC system Äkta Pure 25 from Cytiva. FPLC fractions were analyzed by Coomassie staining and Western blotting, and fractions containing his-tagged proteins were concentrated in Centricon devices, glycerol was added to a final concentration of 50% (v/v) and stored at −80 until their use.

### RT-qPCR

For mRNA expression, Taqman probes for human species were: *ASAH1*, Hs00602774_m1; *ASAH2*, Hs01015658_m1; SMS2, Hs00380453_m1; *SMPD1*, Hs03679347_g1; *SMPD3*, Hs00920354_m1. The RNA was purified using Invitrogen Pure Link RNA miniprep kit, RNA cat. #12183018A, and The cDNA was prepared using Invitrogen SuperScript III First-Strand Synthesis SuperMix for qRT-PCR kit, cat#11752050 following manufacturer’s instructions protocol. All genes were normalized using beta-actin (Hs99999903_m1).

### Transfection

Cells were seeded in 60mm-diameter tissue culture dishes with 1 × 10^6^ cells per dish. The next day, cells were transfected with a final concentration of 20 nM of siRNA using Lipofectamine RNAiMax (ThermoFisher). Media were replaced with fresh media containing 10% FBS after 24h of transfection. Catalog number for siRNA: Life Technology SMPD3 s30927 and s30926, and Qiagen scramble control 1,027,281. GBA2 sequence (25) was cloned in plenti 6.3/V5 DEST vector using gateway cloning.

Plasmid DNAs for sphingolipid metabolizing enzymes were previously published. nSMase2 (26), SMS2 (27), nSMase1 and 3 (28), NCD (4), ASM (29) and ACD (29).

### Lipid measurements

Cells were collected by adding 2 ml of organic solvent (Ethyl acetate: isopropanol 2:3) to the culture dish containing a mix of internal standards (non-natural d17-sphingolipids). The cells were scraped and collected with the organic solvent in glass tubes. Samples were centrifuged for 10 min at 3,000 *g* and the organic phase was transferred to a new glass tube. Cell pellets were subjected to a second extraction with another 2 ml of organic solvent. The combined lipid extract was used for parallel analysis of ceramides, sphingomyelins, and inorganic phosphate. Lipid extracts were evaporated under nitrogen gas in a 45°C water bath. Lipids were resuspended in 150 μl of methanol and stored in mass spectrometry vials (Agilent) at −20°C until they were injected into the LC-MS for their analysis. Samples were analyzed at the Stony Brook Biological Mass Spectrometry Core using a Thermo TSQ Quantiva mass spectrometer equipped with a Vanquish UHPLC system. Flow: 0.5 ml/min; Mobile phase A: Fisher Water Optima LC/MS, 1 mM ammonium formate, 0.2% formic acid; Mobile phase B: Fisher Methanol Optima, 1 mM ammonium formate, 0.2% formic acid. Buffer pre-heated 30°C; Gradient: 0–1 min 80% B, 1–7.0 min 99% B, 7–16 min 99% B. The column was Spectra 3 um C8SR 150 × 3 mmID HPLC Column from Peeke Scientific. Transitions for sphingolipids, retention times, and collision energies were published (30).

### Inorganic phosphate normalization

One ml of the 4 ml of lipid extract was used for inorganic phosphate normalization. The aliquot was evaporated under nitrogen gas at 45°C. Then to the dried sample 1 ml of chloroform, 2 ml of methanol, and 0.8 ml of water were added and vortexed after each addition. An additional 1 ml of chloroform and 1 ml of water were added and samples were vortexed. Samples were centrifuged at 3,000 *g*, for 5 min. The upper phase was discarded by aspiration. The lower phase was recovered into a new glass tube and dried under nitrogen gas.

Phosphate standards were prepared as: 0, 1, 3, 5, 10, 20, 40, and 80 nmol NaH_2_PO_4_. To all samples and standards, 600 μl of Ashing Solution (10N H2SO4: 70% HClO4: H2O) (9:1:40) was added and tubes were vortexed. Pyrex tubes were exposed to 160°C overnight. The following day, 0.9 ml of water was added to all tubes, including standards. This was followed by 500 ul of 0.9% Ammonium Molybdate, and 200 μl of 9% freshly prepared ascorbic acid. Samples were incubated at a 45°C water bath for 30 min, and 200 μl of each sample was transferred into a 96-well plate. Blue color absorbance was measured using a plate reader set at 820 nm. Inorganic phosphate was quantified using the calibration curve built using the standards.

### Immunofluorescence

Cells were grown in gamma-irradiated, 35 mm glass-bottom poly-D-lysine coated dishes (MatTek Corp.; catalog number P35GC-1.5-10-C) at 10^5^ cells/dish. Cells were fixed with 4% paraformaldehyde in PBS for 20 min, labeled with V5 antibody (dilution 1:500, Invitrogen cat. #R96025), probed with the secondary antibody, Alexa Fluor 488 goat anti-mouse (dilution 1:500, Invitrogen cat. # A11001), and visualized using a Leica TCS SP8 confocal microscope with HC PL APO 63×/1.40 oil immersion objective (Morrisville, NC). Leica raw data files (.lif) were generated using Leica Application Suite X, and images were extracted using Bio-formats [OME] (openmicroscopy.org) package for Python (python-formats).

### Western blotting

Cells were collected in 1% SDS (w/v), and, after the addition of Laemmli buffer containing 2-mercaptoethanol, cell lysate was incubated at 100°C for 5 min before being loaded on the gel (BioRad 4%–20% Tris-Glycine gel). After the electrophoresis, proteins were transferred onto a nitrocellulose membrane. The nitrocellulose membrane was blocked for 1 h with 10% Milk in Tris-phosphate buffer saline (TPBS) and then incubated with the primary antibody in 5% bovine serum albumin in TPBS overnight at 4°C. Membranes were washed with TPBS, and incubated for 1 h with appropriate secondary antibody in 10% Milk/TPBS containing HPR enzyme. Western blots were developed with ECL and exposed on X-ray film.

### Software

TraceFinder 4.1 from Thermo was used to quantify sphingolipids from LC-MS raw files. Bibliographic references were managed using Mendeley desktop v 1.19.4 software (mendeley.com) and EndNote 20 (endnote.com). Editing and data processing for this manuscript used free open-source software (FOSS) whenever possible, including Debian Linux (debian.org), LibreOffice (libreoffice.org), GIMP (gimp.org), Python (python.org), Okular (okular.kde.org), and Spyder (spyder-ide.org). GraphPad Prism 9.3.1 was used for plotting the final results for the manuscript (GraphPad Software, graphpad.com).

## Results

### The content in PMCer differs in different cell lines, and it is not correlated to total cellular ceramide

**Fig. 1:**
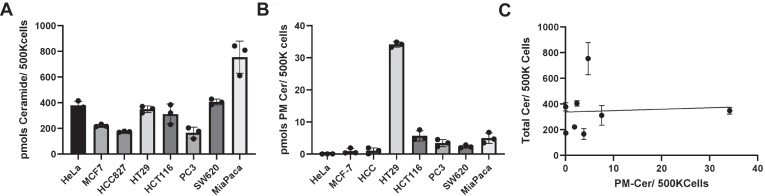
The PMCer basal content in different cell lines is not correlated to total ceramide content. A: Total cellular ceramide content in several cell lines was measured by LC-MS/MS and plotted as nmol of ceramide/500K cells. B: PM-Cer content from the same cell lines. C: Correlation between the total amount of ceramide and PM-Ceramide in the same cell lines. Note: at this cell density, all cell lines were subconfluent.

The recently reported method to measure PMCer (21) allows the characterization of this pool, the dissection of its regulation, and the identification of the metabolic pathways involved. Previously, we quantified the amount of PMCer in HeLa cells, and it represented a very small percentage, nearly 1%, of the total ceramide (21). The amount of PMCer in different cell lines was uncertain, but we hypothesized that it could differ depending on the cell line. To address this point, PMCer was measured in HeLa (Cervical cancer), MCF-7 (luminal breast cancer), MDA-MB-231 (triple negative breast cancer), HCC827 (lung adenocarcinoma), HT29 (colorectal cancer), HCT116 (colorectal cancer), PC3 (prostate cancer), SW620 (colon cancer), and MiaPaca (pancreatic carcinoma) cells. The different cell lines presented distinct levels of total cellular ceramide (Fig. 1A) and even more remarkable differences in PMCer (Fig. 1B). As shown in Fig. 1C, the mass of these two pools of ceramide were not directly related. This suggested that the basal amount of PMCer might not be constant between cell lines, and PMCer levels are not a direct function of total ceramide but are independently regulated.

### An in silico analysis of SL-metabolizing enzyme on PMCer content predicted nSMase2 as the sole enzyme to regulate basal levels of PMCer

**Fig. 2:**
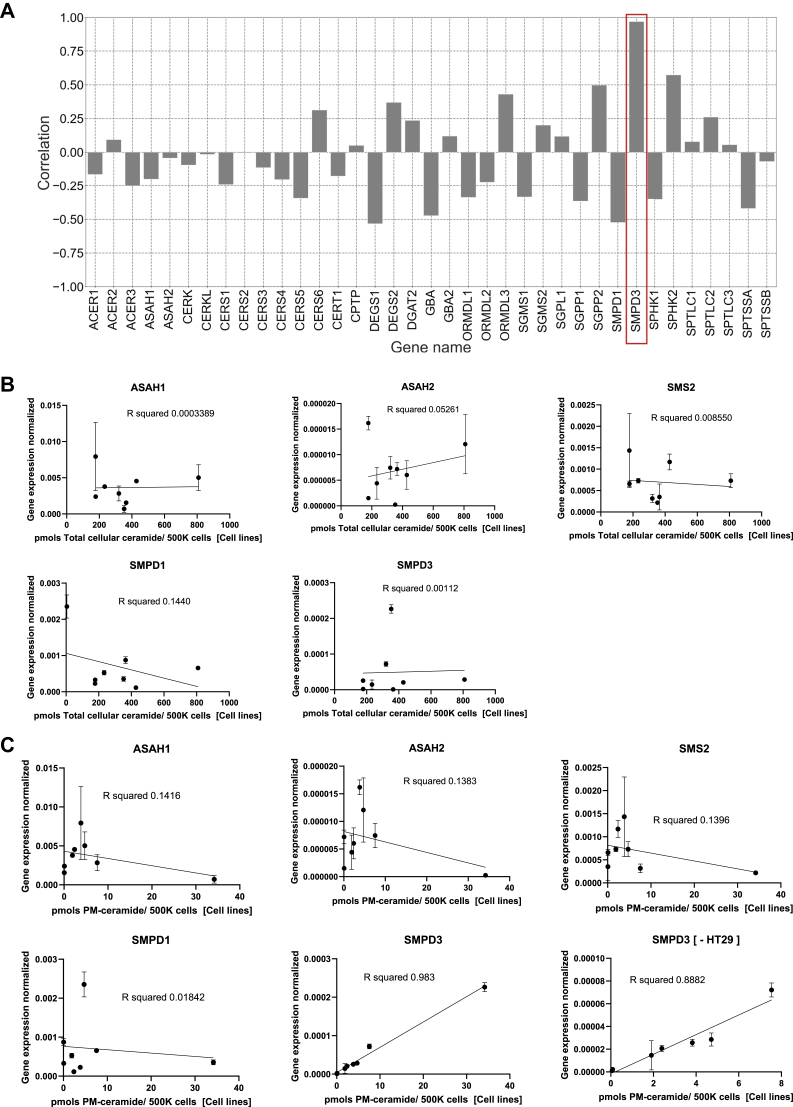
The amount of PMCer tightly correlates with *SMPD3* gene expression levels. A: In silico correlation between PMCer and sphingolipid enzyme expression levels from the CCLE database (using the same cell lines as Figure 1) shows a very high correlation with *SMPD3* (highlighted in *red*). Sphingolipid-metabolizing enzymes are: Alkaline ceramidase 1,2 and 3 (*ACER1*-3), acid and neutral ceramidases (*ASAH1* and 2), ceramide kinase and like-protein (*CERK* and *CERKL*), ceramide synthases 1–6 (*CERS1-6*), ceramide transfer protein (*CERT1*), ceramide-1-phosphate transfer protein (*CPTP*), dihydroceramide desaturase 1 and 2 (*DEGS1* and 2), glucosylcerebrosidase beta 1 and 2 (*GBA* and *GBA2*), sphingolipid biosynthesis regulator 1–3 (ORMDL1-3), sphingomyelin synthase 1 and 2 (SGMS1 and 2), sphingosine-1-phosphate Lyase 1 (*SGPL1*), sphingosine-1-phosphate phosphatase (*SGPP1* and 2), sphingomyelin phosphodiesterase (*SMPD1* and 3), sphingosine kinase 1 and 2 (*SPHK1* and 2), serine palmitoyltransferase long chain base subunit 1 to 3 (*SPTLC1*-3) and serine palmitoyltransferase small subunit A and B (*SPTSSA* and B). B: The gene expression levels for the cell lines were experimentally measured and correlated with the amount of lipids using Pearson correlation. There was no correlation between total ceramide and sphingolipid metabolizing enzyme gene expression. C: When PMCer was used for the correlation studies, only *SMPD3* gene expression levels correlated with PM-ceramide content. To reduce the effect of the cell line with very high *SMPD3* from the analysis, HT29 was removed from the analysis, and the Pearson correlation was still high.

The large differences in PMCer content amongst the different cell lines suggested that sphingolipid metabolizing enzymes may be differently regulated in these cell lines. The Cancer Cell Line Encyclopedia (CCLE) project contains the mRNA expression levels for many cancer cell lines analyzed by RNAseq. To predict what enzymes could regulate the basal levels of PMCer, we correlated the expression levels of each sphingolipid-metabolizing enzyme to the PMCer amount from the same cell lines shown in Fig. 1 (Fig. 2A. The list of sphingolipid metabolizing enzymes can be found in the legend of Fig. 2). Surprisingly, in this in silico prediction, nSMase2 was the only gene with a high correlation with PMCer, whereas other sphingolipid-metabolizing enzymes believed to access the plasma membrane and other enzymes that could supply ceramide indirectly had no correlation or much lower correlation values.

This result was unexpected since many of these enzymes have been reported to regulate PMCer. These include not only nSMase2 (gene *SMPD3*) but also acid sphingomyelinase (ASM, gene *SMPD1*); acid ceramidase (ACD, gene *ASAH1*), and GBA2 for direct generation of PMCer; neutral ceramidase (NCD, gene *ASAH2*) for the clearance of PMCer; and sphingomyelin synthase 2 (SMS2, gene *SGMS2*) with a dual role of clearing and possibly generating PMCer (31, 32).

It is important to mention that independent of their activity on PMCer, these enzymes have also been localized in the plasma membrane. Thus, although ASM is mainly localized in the lysosome, some studies have reported ASM to localize at the plasma membrane directly (33, 34), or having access to the plasma membrane either by the Golgi secretory pathway (29) or by the releasing of the lysosomal content into the cellular medium (35); NCD is mainly localized in the Golgi and the plasma membrane (4, 36, 37), and nSMase activity has been involved in sphingomyelin hydrolysis in response to TNFα at the plasma membrane (38). nSMase2 was also shown to localize in the Golgi and plasma membrane (36). GBA2 is a non-lysosomal-beta-glucocerebrosidase, initially localized in the cell surface (39) but its precise localization remains uncertain. GBA2 has been asymmetrically localized in the extracellular side of the plasma membrane (40). However, this localization has been disputed, and other authors argue that it is exposed to the cytosolic side of the plasma membrane and in the Golgi (41).

**Supplemental Figure 1:**
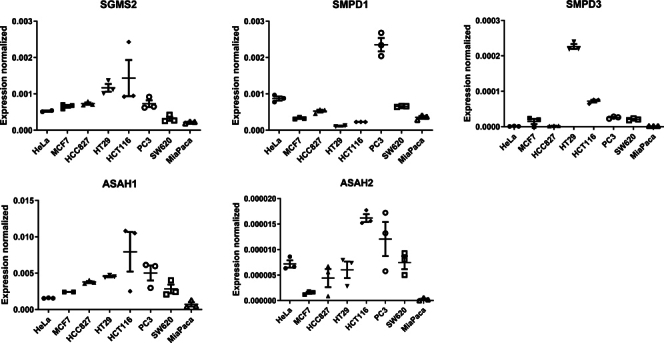
The gene expression level of several SL-metabolizing enzymes in the previously analyzed cell lines was measured using normalized TaqMan probes by RT-PCR.

To evaluate the in silico prediction, the expression levels of nSMase2 mRNA and other key sphingolipid metabolizing enzymes were experimentally measured (supplemental Fig. S1), and the amount of total ceramide and PMCer were quantified under the same experimental conditions in the distinct cell lines. The results suggested that none of the enzymes' mRNA levels correlated significantly with the total amount of ceramide (Fig. 2B). This supports the idea that various enzymes contribute discrete ceramide pools to the total ceramide levels. However, when the amount of PMCer was used for the correlation (Fig. 2C), *SMPD3* had the best correlation with PMCer and showed an almost perfect correlation, with a Pearson R square value of 0.98.

These results suggested that the endogenous expression levels of nSMase2 drive the PMCer content in all cell lines tested, at least in basal, unstimulated conditions.

### Overexpression of nSMase2, but not other ceramide-generating enzymes, markedly increased the levels of PMCer

The previous results suggested that the steady-state levels of PMCer depend on nSMase2, and the endogenous mRNA levels determine the amount of PMCer. To evaluate this role further, the effect of upregulation of sphingolipid metabolizing enzymes on PMCer was evaluated.

**Fig. 3:**
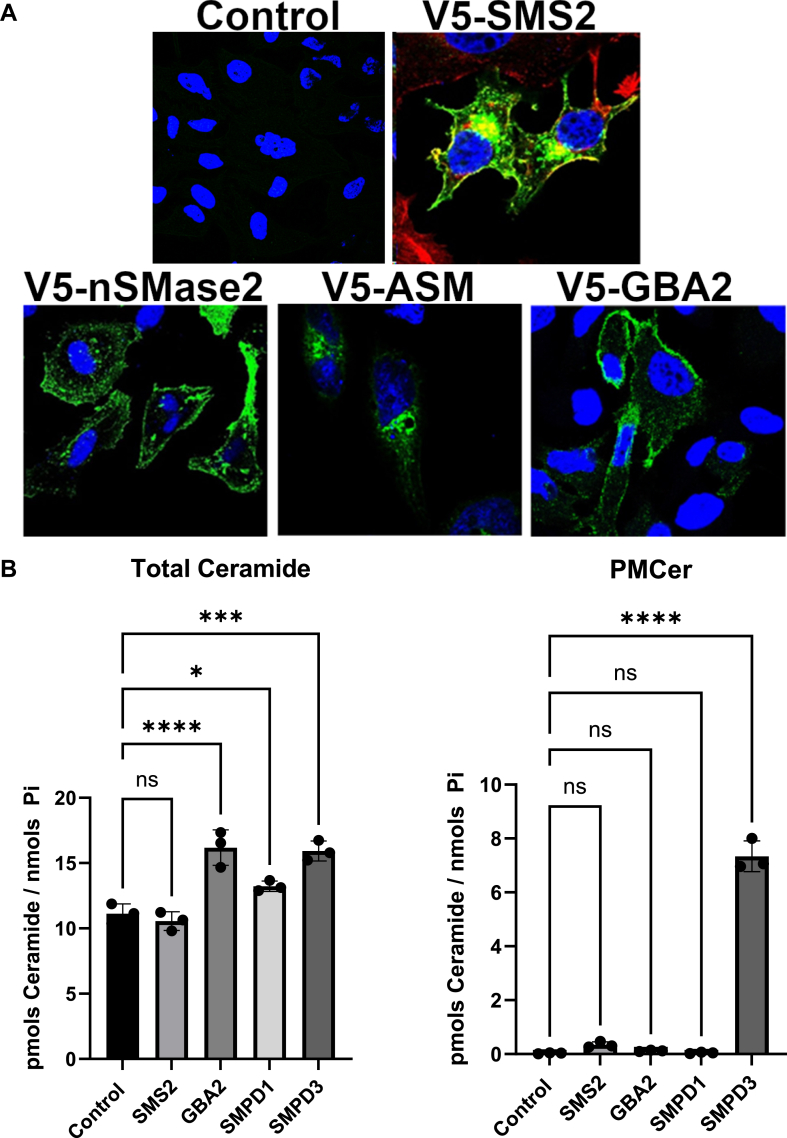

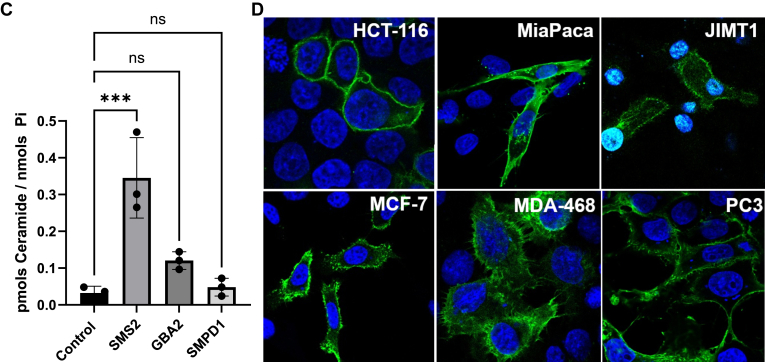

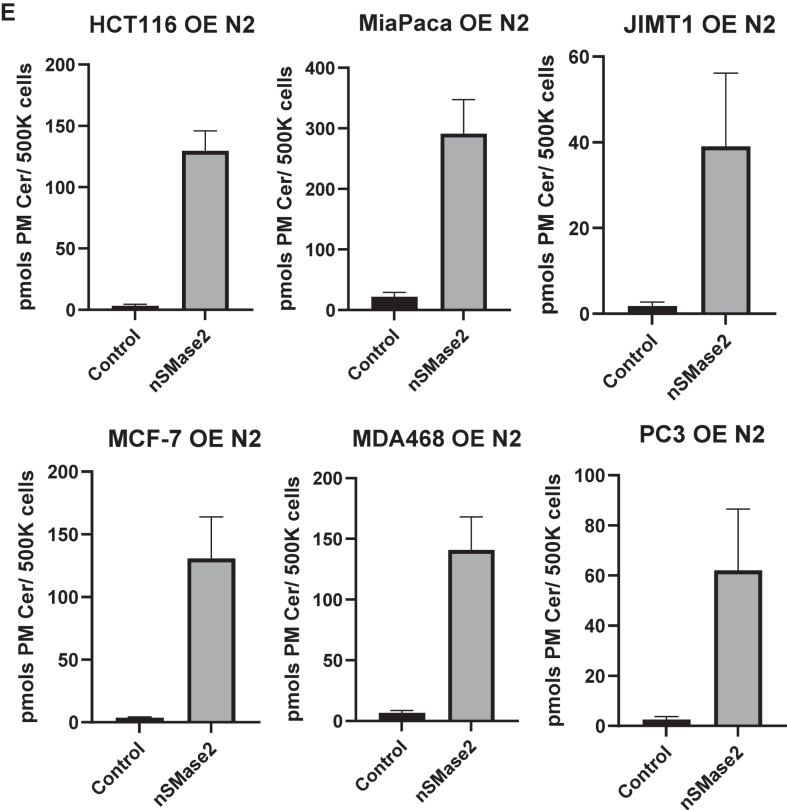
Overexpression of SMPD3 but no other ceramide-generating enzymes with access to the plasma membrane generated PMCer. A: Subcellular localization of ceramide-generating enzymes with plasma membrane and extracellular media localization. All enzymes were V5-tagged, transfected in HeLa cells, and visualized using a confocal microscope (*Green* V5, *Blue* DAPI to stain the cellular nucleus). B: The total content of ceramide was measured in mock-transfected cells (Control) and cells overexpressing the different constructs. Ceramide was calculated as pmols ceramide/nmol Pi. C: Same cells were used to calculate the amount of PM-Cer. An insert excluding nSMase2 shows the effects of SMS2, GBA2, and SMPD1 on PMCer. D: nSMase2 (V5-tagged, green) was overexpressed in different cell lines. E: A dramatic elevation of PMCer was observed in all cell lines tested (HCT116, Mia Paca, JIMT1, MCF-7, MDA231, and PC3). Statistics: One-way ANOVA. ∗*P* < 0.05, ∗∗*P* < 0.01, ∗∗∗*P* < 0.001. ∗∗∗∗*P* < 0.0001.

**Supplemental Figure 2:**
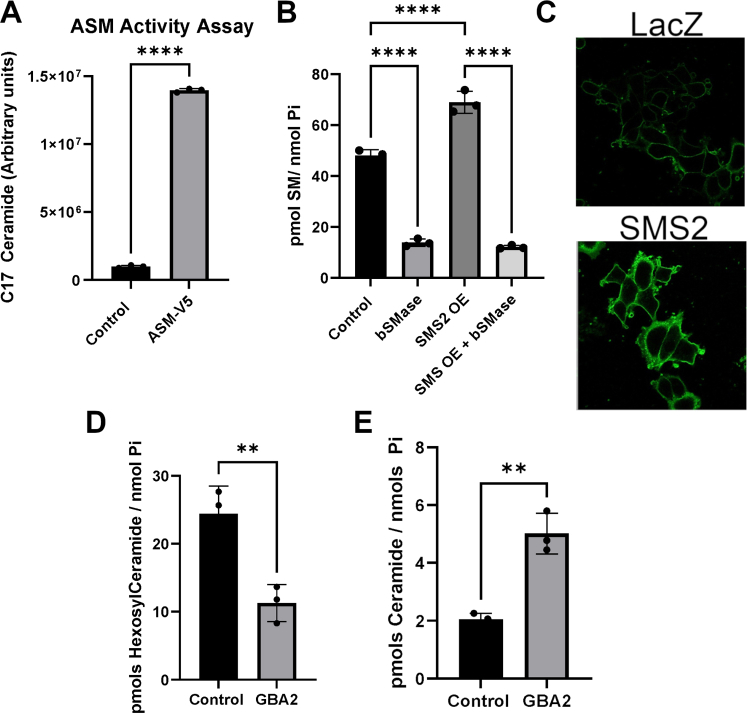
Validation of transfected ASM, SMS2, and GBA as active constructs. (A) Acid Sphingomyelinase activity assay of media of cells overexpressing ASM, showing ASM-V5 was expressed and secreted to the media, with potential access to the plasma membrane. (B) Overexpression of SMS2 (SMS OE) increased cellular sphingomyelin. Bacterial sphingomyelinase (bSMase) drops sphingomyelin to the same level suggesting the increase of SM it is in the plasma membrane. (C) Lysenin, a sphingomyelin binding protein, showing SMS2 increased sphingomyelin at the plasma membrane. (D) Overexpression of GBA2 decreased cellular hexosylceramide. (E) and increased total ceramide.

We overexpressed several ceramide-generating enzymes in HeLa cells. This cell line was selected since it is easily transfected at high efficiency and has a low PMCer level. Included in the study were nSMase2 and all other enzymes reported or suggested to be able to directly generate PMCer based on their subcellular localization at the plasma membrane (these were nSMase2, SMS2, ASM, and GBA2). Figure 3A shows the subcellular localization of the overexpressed enzymes carrying a tag used for immunostaining. Confirming previous literature, nSMase2, GBA2, and SMS2 showed a clear plasma membrane localization (36). ASM was tagged at the C-terminal with a V5 tag and showed the expected intracellular localization (29). However, it is reported that ASM is also secreted into the media, with access to the plasma membrane. To ensure that the lysosomal ASM was secreted into the media, activity assays were performed towards liposomal sphingomyelin in cell lysates and extracellular media, showing that the expressed ASM was active in the cells as well as in the media (supplemental Fig. S2A).

Individual expression of each enzyme modulated total cellular ceramide, as previously reported. Overexpression of SMS2 did not affect cellular ceramide but dramatically increased the sphingomyelin content at the plasma membrane (Fig. 3B, and supplemental Fig. S2B, C). ASM and nSMase2 increased the total cellular content of ceramide (Fig. 3B). Remarkably when PMCer was measured, nSMase2 had both a dramatic and a statistically significant effect on PMCer (Fig. 3B). When nSMase2 was removed from the statistical analysis, SMS2 had a modest but statistically significant effect on PMCer (Fig. 3C). GBA2 expression also increased PMCer, but this did not reach statistical significance. The cellular activity of GBA2 was demonstrated by finding approximately a 50% reduction of the content in hexosylceramide and an increase in the total ceramide levels (supplemental Fig. S2D, E). This result suggested that from all claimed enzymes capable of working on PMCer, only nSMase2 had a high capacity to induce its levels.

Although nSMase2 has been widely studied, the localization of this enzyme has only been validated in a few cell lines (36, 42). Thus, nSMase2-V5 was expressed in several cell lines, and the expression and localization of nSMase2 were evaluated (Fig. 3D). In all tested cell lines, overexpression of nSMase2 localized at the plasma membrane and dramatically increased the amount of PMCer (Fig. 3E).

### Overexpression of ceramidases decreased intracellular ceramide but did not decrease PMCer

**Fig. 4:**
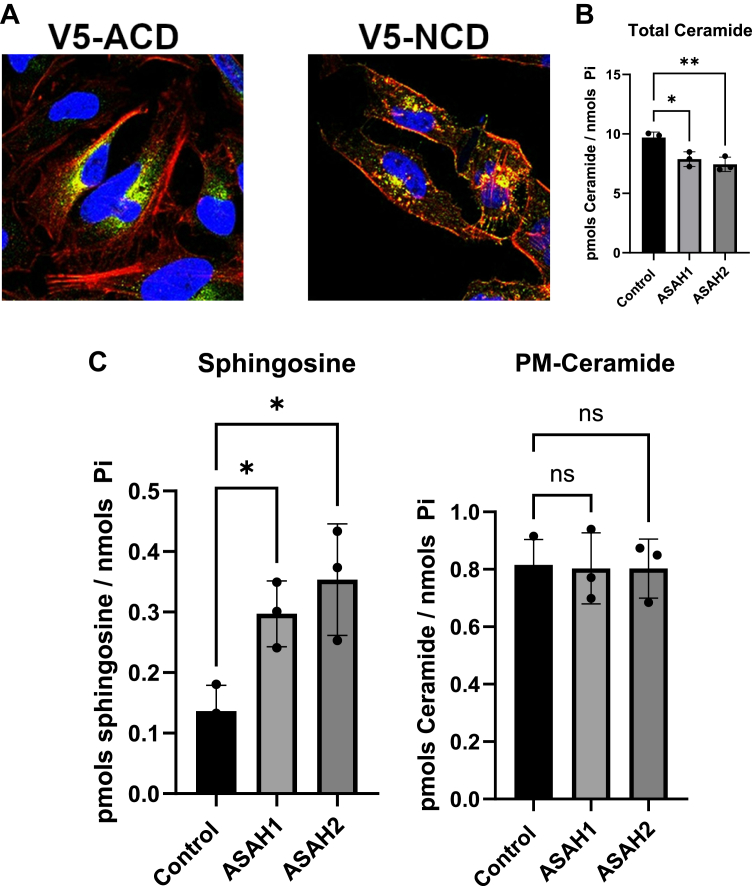
Overexpression of cellular ceramidases with access to the plasma membrane and extracellular media did not reduce PMCer. A: Visualization of V5-tag expression, revealing the intracellular localization of V5- ACD and, also plasma membrane localization of V5-NCD (*Green*: V5 tag, *Red*- Cortical cytoskeleton; *Blue* DAPI nuclear stain). Lipids measurements for (B) total Ceramide, (C) sphingosine, and (D) PM-Ceramide. Statistics: One-way ANOVA. ∗*P* < 0.05, ∗∗*P* < 0.01.

**Supplemental Figure 3:**
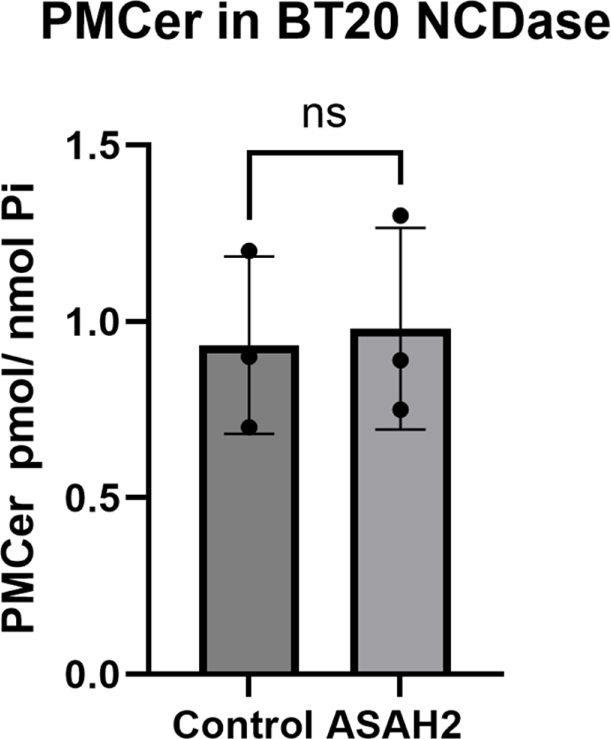
Quantification of PMCer in BT20 cells transfected with empty vector or ASAH2 plasmid.

The levels of PMCer must be regulated not only by its production but also by its clearance. Two ceramidases would potentially have access to PMCer, NCD, and ACD, to hydrolyze PMCer into sphingosine. Tani *et al.* demonstrated that PMCer was metabolized by NCD at the plasma membrane to generate sphingosine and, eventually, sphingosine 1-phosphate (43). The previous experiments evaluating the PMCer anabolic enzymes were done on cell lines with low content of PMCer, and therefore, a decrease of PMCer in HeLa cells with low basal levels of PMCer could be difficult to detect. Hence, NCD and ACD were evaluated in HT29 cells (Fig. 4A), which have a high content in PMCer. Cells overexpressing NCD and ACD showed a decrease in the amount of cellular ceramide (Fig. 4B), and an increase in sphingosine (Fig. 4C), suggesting that both ceramidases were active in the cell. Neither overexpressed ceramidase resulted in major changes in PMCer (Fig. 4D). We tested a different cell line to rule out the possibility of a cell line effect on NCD. According to the CCLE database, BT20 contains a very high level of expression of nSMase2, and RT-PCR of this cell line confirmed a similar level to that of HT-29 and very high levels of PMCer. Overexpression of NCD in BT20 also changed total ceramide and sphingosine levels but did not affect the amount in the plasma membrane (supplemental Fig. S3).

These results suggest that even if NCD can hydrolyze PMCer to form sphingosine, the kinetics must be much slower than the effects of nSMase2 in replenishing the amount of PMCer in a way that the effects of NCD on the PMCer are minimal.

### Only nSMase2 and not other neutral sphingomyelinases generated PMCer

Although nSMase2 is the most studied neutral sphingomyelinase in the cell, other enzymes have been shown to have neutral sphingomyelinase activity. In humans, nSMase1 (gene: *SMPD2*), 2, and 3 (*SMPD4*) have been reported. Not much is known about nSMase1, and 3 in their role as sphingomyelinases, and published data have raised doubt about whether they act as sphingomyelinases in cells. In one publication, nSMase3 did not show neutral sphingomyelinase activity in vitro or in vivo (28). However, other publications have claimed sphingomyelinase activity in cells (44). One study implicated nSMase1 in the formation of ceramide microdomains during the formation of exosomes (45), and nSMase3 has been proposed as specific for striated muscle. In the same work, nSMase3 was reported to localize in the mitochondria, the ER, and “near to the plasma membrane”, with a potential role in generating PMCer (44).

**Fig. 5:**
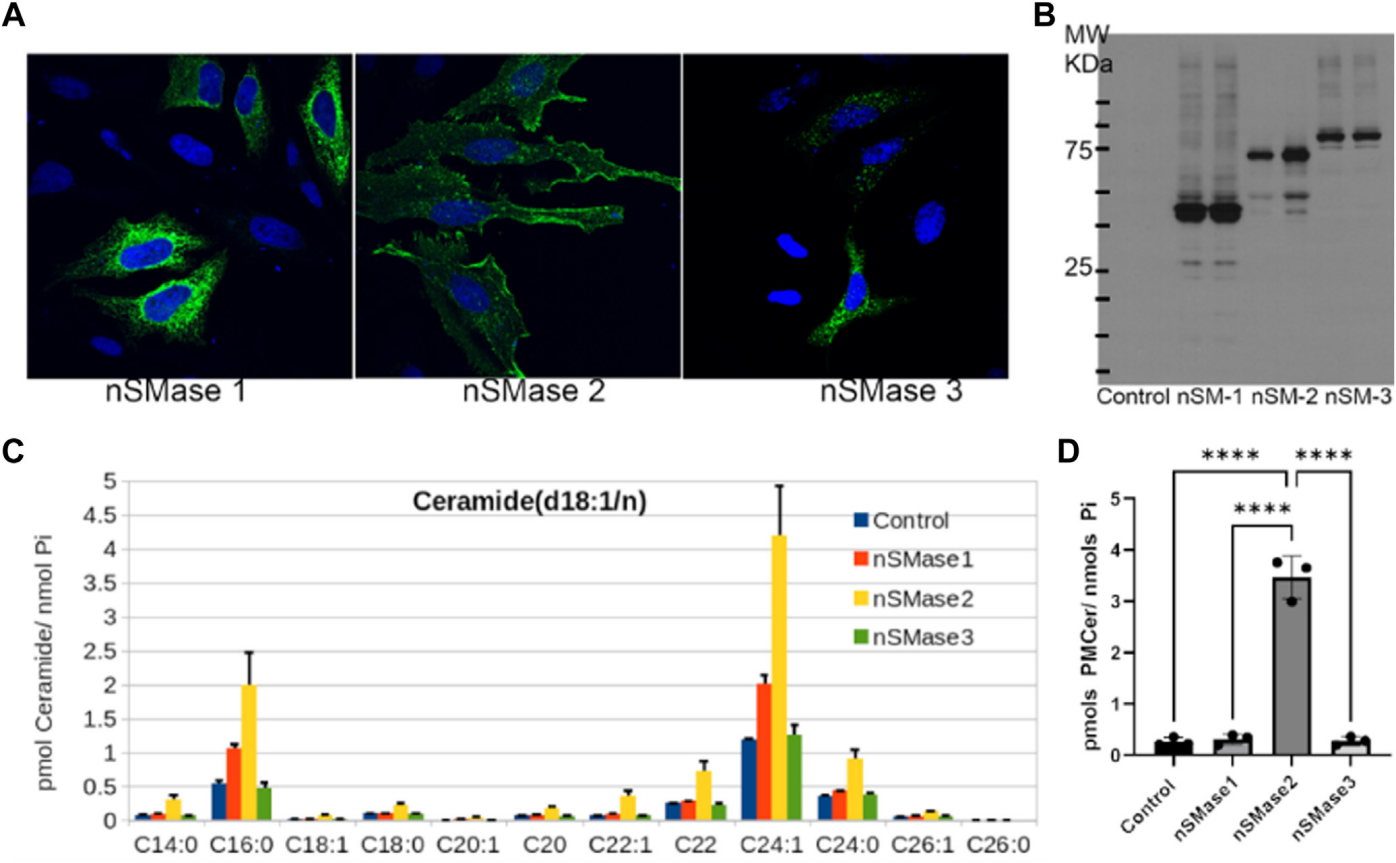
From all neutral sphingomyelinases, only nSMase2 affects PMCER. HeLa cells were transfected with cDNA for empty vector and V5-tagged nSMase1,2 and 3. A: Confocal microscopy images showing expression and localization of the constructs. Only nSMase2 showed a clear plasma membrane localization. B: Western blotting of cell lysates against V5- antibody. Biological duplicates are shown for each condition. Each construct shows protein expression at the correct molecular weight. C: Lipidomics Ceramide profile from the transfected cells, and (D) PMCer quantification. Statistics: One-way ANOVA. ∗∗∗∗*P* < 0.0001.

Using tagged constructs for these enzymes, overexpressed nSMase1 and 3 did not localize at the plasma membrane of HeLa cells (Fig. 5A, B shows a Western blot confirming the expression and size of each enzyme). nSMase2 increased the levels of total ceramides substantially, and nSMase1 produced a much smaller elevation, suggesting that nSMase1 may also act as sphingomyelinase in cells but in a more limited fashion. On the other hand, nSMase3 did not alter the ceramide profile in the cells (Fig. 5C). Importantly, nSMase2 could increase PMCer, whereas nSMase1 and 3 had no effect (Fig. 5D). Therefore, from the 3 gene products of known neutral sphingomyelinases, only nSMase2 localized at the plasma membrane and could generate PMCer.

### Genetic suppression of nSMase2 depletes PMCer

**Fig. 6:**
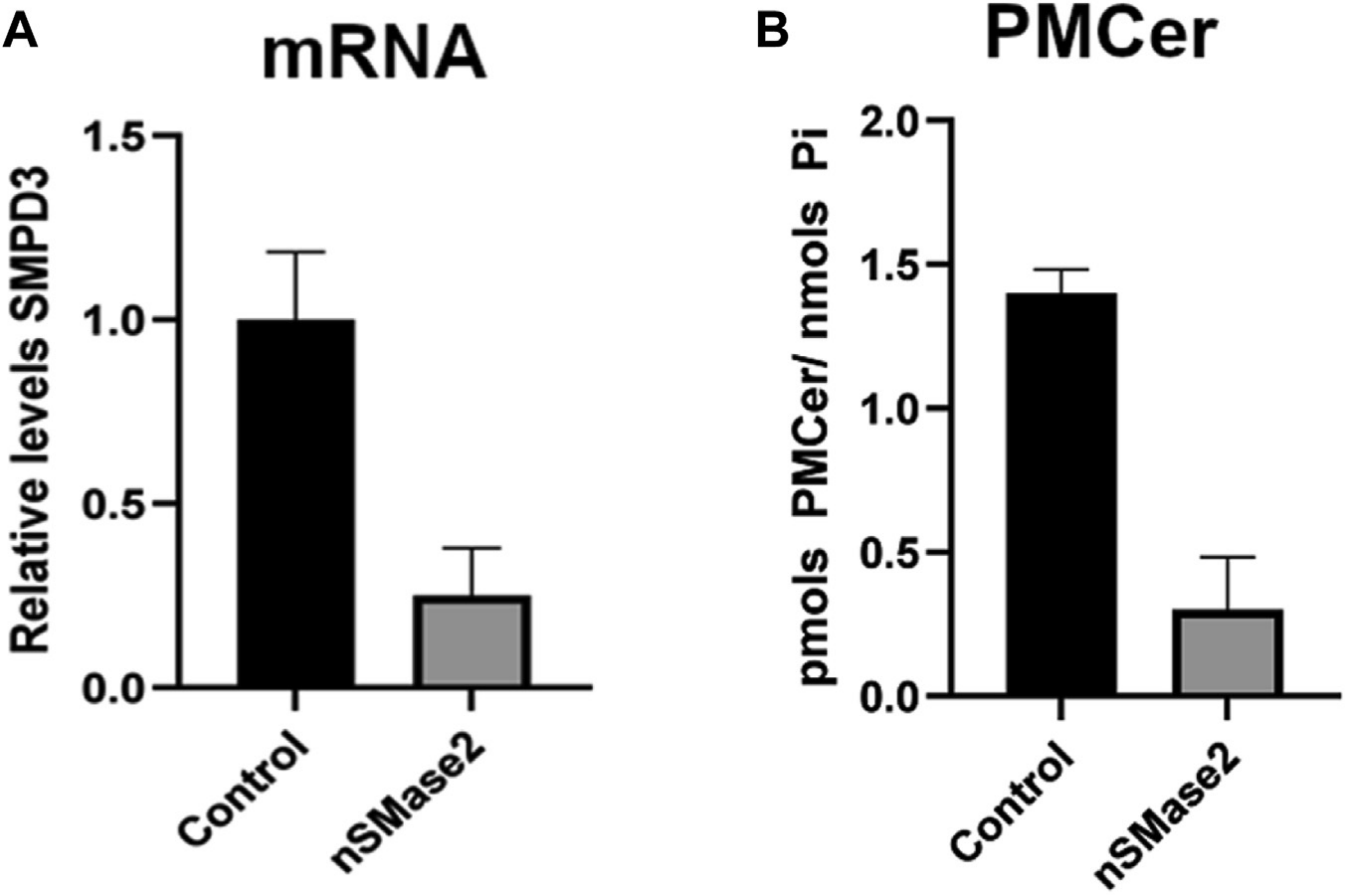
Knockdown of nSMase2 reduces PM-ceramide in HT29 (High SMPD3). HT29 cells, naturally containing high levels of nSMase2 and PMCer, were knocked down for SMPD3. A: mRNA for SMPD3 in control and knocked down cells. B: Quantification of PMCer by LC-MS. Statistics: *t* test.

The previous results showed that nSMase2 was sufficient to generate PMCer. However, it was still possible that other enzymes contributed to the amount of PMCer. To investigate whether nSMase2 was necessary for PMCer, we knocked down nSMase2 and evaluated the amount of PMCer (Fig. 6). HT29 cells, which have a high content in PMCer, were used to knock down nSMase2. Downregulation of nSMase2 led to a dramatic reduction of PMCer, suggesting that nSMase2 is the main enzyme required to form steady-state levels of PMCer (Fig. 6A, B, respectively).

### Confluence generates PM-Cer through nSMase2 expression

**Fig. 7:**
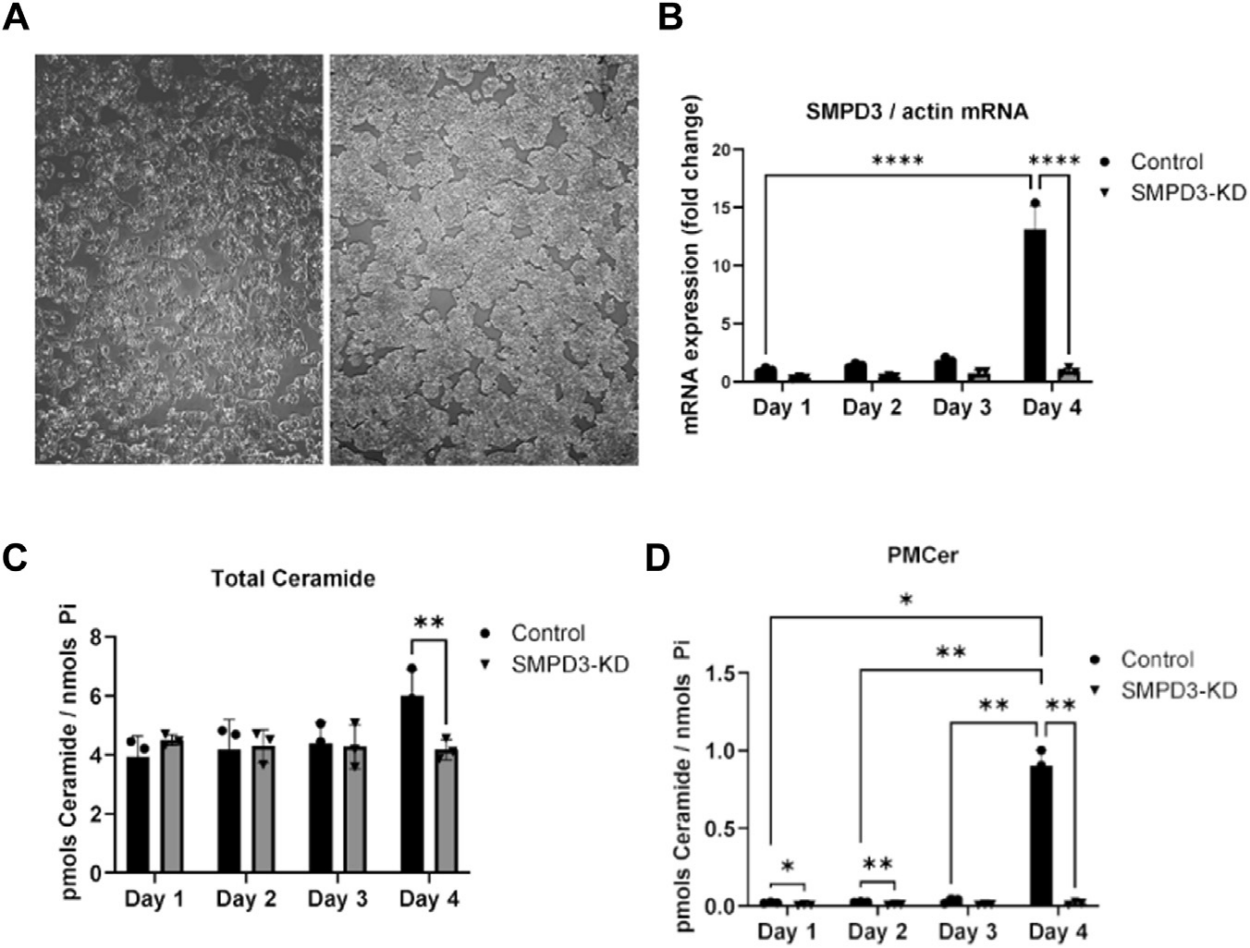
Increase of nSMase2 mRNA expression by confluence results in an increase in PMCer in HT-29 cells. A: HT-29 cells were grown for several days (1–4), increasing confluence. B: SMPD3 mRNA increased with confluence. Knockdown of SMPD3 was effective and blocked SMPD3 increase even at confluency. C: The total levels of cellular ceramide also increased by around 30% with confluency. D: PMCer dramatically increased from almost undetectable to levels that account for the increase in total ceramide. Statistics: One-way ANOVA. ∗*P* < 0.05, ∗∗*P* < 0.01, ∗∗∗∗*P* < 0.001.

**Supplemental Figure 4:**
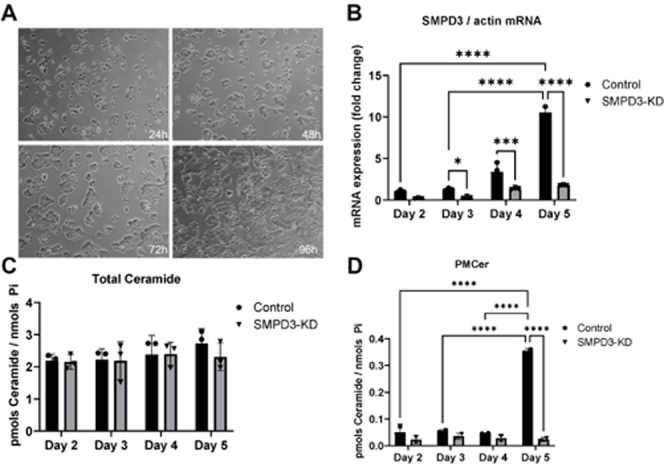
Increase of nSMase2 mRNA expression by confluence results in an increase in PMCer. (A) MCF-7 cells were grown for several 5 days increasing confluence as reported previously (B) SMPD3 mRNA increased with confluence. Knockdown of SMPD3 was effective and blocked SMPD3 increase even at confluency. (C) The total levels of cellular ceramide showed a tendency to increase, but statistics were not significant. (D) PMCer dramatically increased dramatically parallel to SMPD3. Statistics: One-way ANOVA. ∗∗∗∗*P* < 0.0001.

The previous data showed that nSMase2 correlates with PMCer, and changes in nSMase2 produced by overexpression or knockdown regulate PMCer where other enzymes failed. These results opened the question of whether biological events resulting in changes in the levels of nSMase2 would also increase PMCer. One of the first and well-established biologies increasing nSMase2 is cell confluence in MCF7 cells (26). We tested MCF-7 and HT29 cells. Cells were plated at low confluence, and after a few days, they reached confluence (Fig. 7A for HT-29, and supplemental Fig. S4A for MCF-7). The levels of nSMase2 increased in both cell lines as the cells reached confluence (Fig. 7B, and supplemental Fig. S4B). As previously published, the levels of total ceramide increased by around 10%–20%, and this increase was dependent on nSMase2 (Fig. 7C, and supplemental Fig. S4B). Notably, when PMCer was measured, the almost undetectable PMCer dramatically increased with confluence several tens of fold in HT-29, and more modest, but also highly statistically significant in MCF-7 cells (Fig. 7D, and supplemental Fig. S4D). Interestingly, in HT-29 cells, the amount of PMCer increase (∼1pmol/nmol Pi) accounted for a large part of the amount of total ceramide (∼1.5–2 pmol/nmol Pi) induced by nSMase2. These results show that PMCer is tightly regulated by nSMase2, and changes in expression levels translate to significant changes in PMCer.

## Discussion

A growing number of studies support the idea that the biological functions of ceramide must be mediated by distinct pools located in different subcellular membranes (1, 10, 46). Plasma membrane ceramide has emerged as a bioactive messenger sufficient to promote loss of cell adhesion and increase migration. Existing methodology has used diverse ceramide antibodies to detect changes in PMCer. However, the amount of PMCer in cells could not be quantified. This lack of methodology prompted the development of a novel method to quantify PMCer, which, in this work, allowed us to quantify, for the first time, the basal levels of PMCer and identify nSMase2 as a main enzyme to regulate them in resting cells.

Cells contain several sets of enzymes to generate ceramide. These are often present in several subcellular locations, making determining their site of action difficult. Here we have shown that from all known enzymes with access to the plasma membrane, only nSMase2 proved to be able to regulate steady-state PMCer levels. This is notable since these enzymes are located at least partially in the plasma membrane and show cellular activity. For example, overexpression of GBA2 dramatically reduced hexosylceramides, suggesting that large levels of PMCer should be produced. Indeed, a large amount of total cellular ceramide is generated, but no significant levels of PMCer were detected. Either this ceramide is not generated at the plasma membrane, it is rapidly metabolized, or it is transported to other membranes.

Many of our results converged to demonstrate that nSMase2 was the main ceramide-metabolizing enzyme that regulates PMCer. Thus, (i) we have shown a striking near-perfect correlation between the endogenous mRNA levels and PMCer content. (ii) Overexpression of nSMase2, but not other sphingolipid enzymes, dramatically increased PMCer. (iii) Knockdown of endogenous nSMase2 depleted PMCer. (iv) Only nSMase2 resulted in substantial PMCer regulation from all reported human-neutral sphingomyelinases. It is important to note that 1) nSMase2 might also be generating ceramide in other compartments besides the plasma membrane (as shown in Golgi (36), lipid droplets (42), and putatively in the nucleus (47)), and 2) We have studied the regulation of PMCer in unstimulated cells. It is possible that certain stimuli could regulate nSMase2 activity through post-translational modifications modulating enzymatic kinetics and/or through modulating its subcellular distribution and, thus, conditionally regulating PMCer levels (42, 48, 49). Moreover, we do not discard the fact that other enzymes of ceramide metabolism might also generate PMCer under specific conditions. However, this might need a more complex regulation of these enzymes (for example, through post-translational modifications, co-factors, or membrane trafficking) since increasing messenger levels of these enzymes did not result in PMCer generation. In this sense, overexpression of both SMS2 and GBA2 were capable of increasing PMCer. However, this increase was minimal when compared to nSMase2 overexpressing cells. Although both enzymes showed cellular activity towards their known natural substrates, it is possible these were not the optimal conditions for these enzymes to act on PMCer. In the case of SMS2, we showed that its overexpression also increased plasma membrane sphingomyelin, suggesting that SMS2 is acting as sphingomyelin synthase. In this case, PMCer must probably result from a compensatory reaction more than the reverse activity of this enzyme. Anti-ASM antibodies have been shown to co-localize with anti-ceramide antibodies at the plasma membrane, and the authors have suggested that ASM directly generates PMCer (50). However, in our study overexpression of ASM, and detection of active form in the cell culture media, did not increase PMCer, suggesting that the presence of ASM with access to the plasma membrane is not sufficient to generate PMCer. However, we do not want to discard that ASM might act on the plasma membrane under different conditions, upon the presence of co-activators or specific cell lines. Although, according to the current models, ASM acts on the outer leaflet and nSMase2 on the inner leaflet of the plasma membrane, the methodology employed should measure the whole amount of ceramide in the plasma membrane (21). Moreover, PMCer might be modulated by intra-cellular membrane transport or exosome formation. We also contemplate the possibility that PMCer might be modulated by intra-cellular membrane transport or exosome formation. Ceramide generated by nSMase2 or other enzymes might be transported to or from the plasma membrane. This might also be an important factor in PMCer regulation that should be incorporated into the PMCer equation in future work. The results of this work imply that under basal conditions, mRNA levels of nSMase2 are the main driving force in the regulation of PMCer.

PMCer is a source of biologically active S1P, regulated by NCD. Our data support the idea that when PMCer is increased, NCD produces sphingosine, which can then be further metabolized into S1P. This observation suggests that NCD would also reduce the amount of PMCer, regulating, then, its levels. However, since the amount of S1P is very low, a small amount of hydrolysis of PMCer will generate a biologically significant level of S1P (a few nM is bioactive) but does not appreciably affect the total level of PMCer. The action of nSMase2 can easily overcome any effect of NCD, producing bioactive sphingosine and S1P without affecting the amount of PMCer.

Physiological elevation of nSMase2 by cells reaching confluence also resulted in a parallel increase in total cellular ceramide and PMCer production. Assessing the changes in total ceramide generated or metabolized by individual pathways is difficult. However, the total amount is the arithmetic result of these individual changes and locations. In contrast, the amount of PMCer might reflect the result of one single enzyme and one location. During confluence, the increase on PMCer is not trivial, and it accounts for a large percentage of the net increase in total ceramide, suggesting that nSMase2 and PMCer are the main contributors to the detected increase in total ceramide. This significant contribution of the nSMase2 response also suggests that PMCer might be tightly regulated in known nSMase2-mediated biologies. For example, PMCer could play a role in cell adhesion, migration, cell death, or lipid droplet formation. Moreover, these results also prove that quantifying PMCer is a powerful tool to measure nSMase2 activity in the cell. Where many distinct enzymes control total ceramide measurement, PMCer is free of this cellular interference, allowing a more direct evaluation of nSMase2 activity. For example, the PMCer assay could be used to evaluate new nSMase2 inhibitors *in cellulo*, or gene connections to nSMase2.

Another important point to mention is that when SMPD3 mRNA is elevated, the amount of PMCer is very close to the increment of total ceramide. This could suggest that in unstimulated conditions, nSMase2 works mainly at the plasma membrane, with little action on other compartments.

Many of the publications on PMCer have focused on cancer. Thus, all cell lines tested in this study were established commercially available cancer cell lines. Although we tried to represent cell lines with different tissue origins, it is possible that some tissue-specialized cells might have other mechanisms to regulate PMCer.

In conclusion, this work shows that of the enzymes known to modulate the levels of ceramide directly, many of them known to localize, at least partially, in the plasma membrane, only nSMase2 regulates the PMCer in cells, at least in unstimulated conditions. This regulation is tightly associated with nSMase2 transcriptional levels and opens the question of whether many of the biological functions related to nSMase2 are regulated, at least in part, by PMCer.

## Data availability

Mass spectrometry data will be shared upon request: Daniel Canals, Stony Brook University, Cancer Center. MART building. Daniel.Canals@stonybrookmedicine.edu.

## Supplemental data

This article contains supplemental data.

## Conflict of interests

The authors declare that they have no conflicts of interest with the contents of this article.
